# Gross and Histological Examination of a Large Spheno-Orbital Meningioma

**DOI:** 10.7759/cureus.10256

**Published:** 2020-09-05

**Authors:** Anna E Kaiser, Sriya V Reddy, Matthew A Von Zimmerman, Amber Gordon, Francis J Liuzzi

**Affiliations:** 1 Medicine, Lake Erie College of Osteopathic Medicine, Bradenton, USA; 2 Anatomy, Lake Erie College of Osteopathic Medicine, Bradenton, USA

**Keywords:** spheno-orbital meningioma, meningothelial meningoma

## Abstract

Meningiomas arise from arachnoid cap cells and are the most common heavily researched intracranial tumors. Most of these neoplasms are benign and are classified as World Health Organization (WHO) grade I. They are often found in parasagittal and falx regions, over cerebral convexities, and in the sphenoid ridges. Spheno-orbital meningiomas (SOMs) occupy the cranium and the orbit and are less commonly encountered. Nonetheless, in this case study, a 9.5 cm × 5 cm SOM occurring in a 93-year-old female cadaver was identified and examined. The tumor spanned from the left middle cranial fossa, through the anterior fossa and invaded the orbit. It caused proptosis of the left eye, compression of the temporal lobe, and damage to the optic nerve. Histological examination of the tumor revealed characteristics of a WHO grade I meningothelial meningioma.

## Introduction

Meningiomas are the most common primary brain tumors accounting for 24%-37.9% of intracranial neoplasms [1,2]. According to the WHO, the majority of meningiomas are benign and are categorized as grade I [2]. Some of these benign subtypes include meningothelial, fibrous, transitional, and psammomatous meningiomas, and they carry a low risk of recurrence and aggressive growth. However, more invasive meningioma subtypes with a high risk of recurrence include atypical and anaplastic, which are grade II and III neoplasms, respectively.

Meningiomas arise from arachnoid cap cells of the meninges and occur predominantly in middle-aged and elderly patients, peaking in the sixth and seventh decades of life [2]. Childhood tumors are less common but tend to be more aggressive. Some risk factors for meningiomas include genetic predispositions, radiation exposure, and the female gender [3,4]. Genetically, meningiomas are a key feature of neurofibromatosis type 2 (NF2), which are linked to mutations on chromosome 22 [2]. Mutations in the *NF2* gene are associated with 30%-70% of all meningiomas; however, additional loci have been identified on chromosomes 18, 1, 14, 10, 9, and 17 [3].

There is a marked female bias for intracranial meningiomas in middle-aged patients with a female to male ratio of 1.7:1. Approximately 90% of spinal meningiomas occur in females [2]. There is evidence that suggests a role for sex hormones in meningioma growth. Many of these tumors display a combination of estrogen, progesterone, and androgen receptors, leading to increased growth during pregnancy and the luteal phase of the menstrual cycle [3]. Targeting these receptors has caused antiproliferative effects and has been shown to be a viable treatment option in in vitro studies.

The majority of meningiomas arise in intracranial, intraspinal, or orbital locations [2]. Intracranially, the most common sites include cerebral convexities, olfactory grooves, sphenoid ridges, para/suprasellar regions, optic nerve sheath, petrous ridges, tentorium, and posterior fossa [2]. It has been reported that only 9%-18% of intracranial meningiomas are spheno-orbital in location [5,6]. Meningiomas involving the orbit can be classified as primary orbital, originating from the optic nerve sheath or from the orbital face of the sphenoid bone, or as secondary orbital, originating from the inner or outer sphenoid ridge, from the tuberculum sellae or from the paranasal sinuses [7].

Because of the rarity of spheno-orbital meningiomas (SOMs), it is necessary that additional research on these tumors be conducted and reported. The current case describes a remarkably large SOM occurring in a 93-year-old cadaver who likely spread from the middle cranial fossa anteriorly through the superior orbital fissure into the orbit.

## Case presentation

This report describes an unusual case of a 93-year-old female cadaver with a large SOM occupying the left middle cranial fossa and invading the left orbit. Her cause of death was listed as blunt force trauma of which we found no evidence.

**Figure 1:**
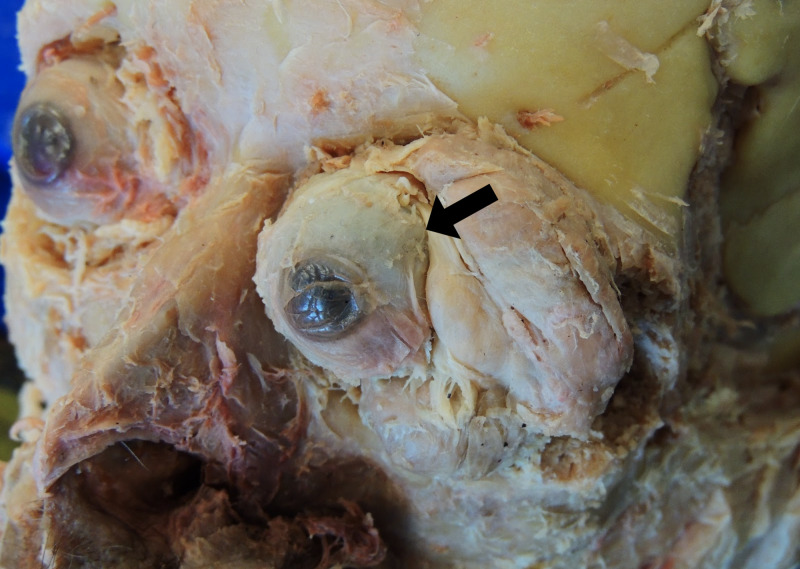
The left eyeball displays proptosis (arrow) due to anterior pressure of the meningioma occupying the left orbital region.

**Figure 2:**
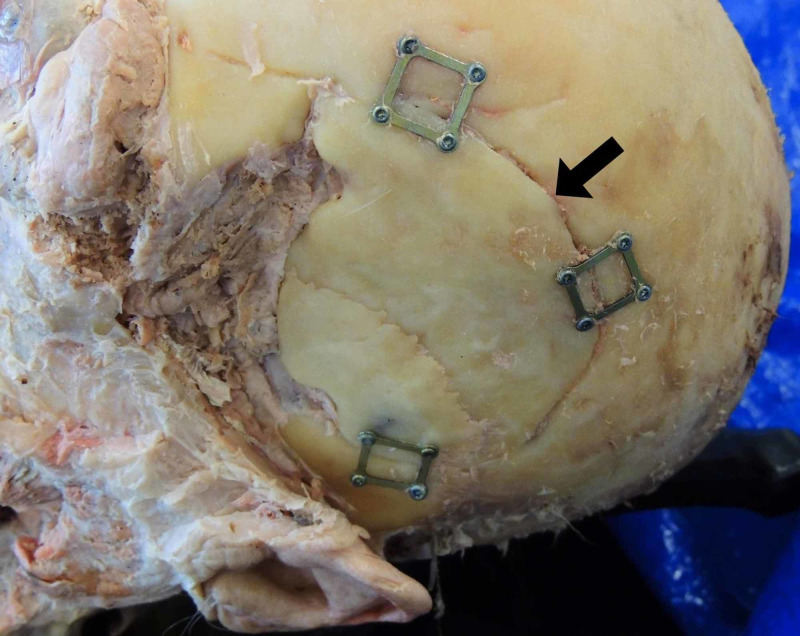
The surgical site (arrow) of a previous craniotomy in a 93-year-old cadaver.

**Figure 3:**
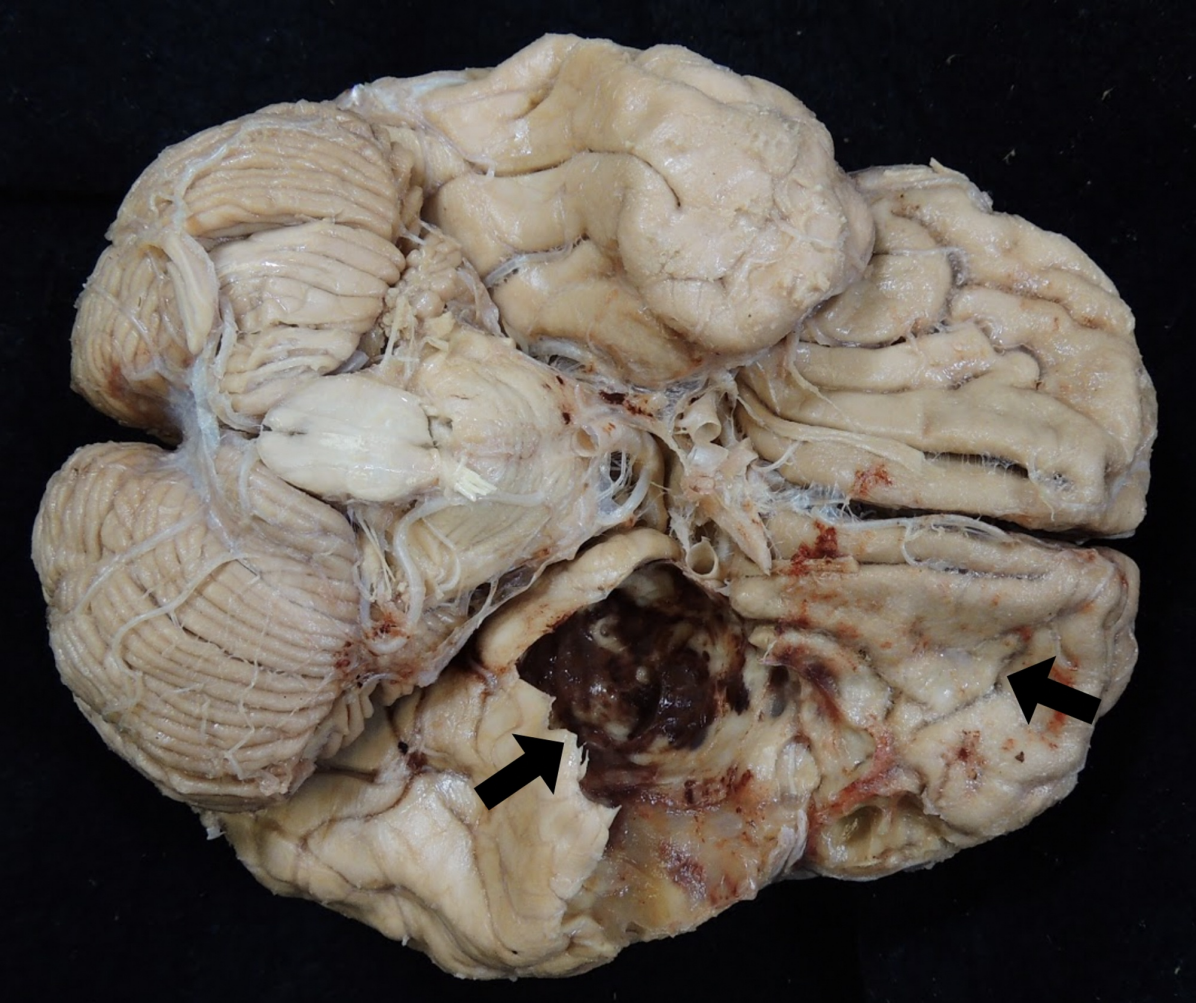
The meningioma severely compresses the left temporal lobe (left arrow), and there is mild compression in the left frontal lobe (right arrow).

**Figure 4:**
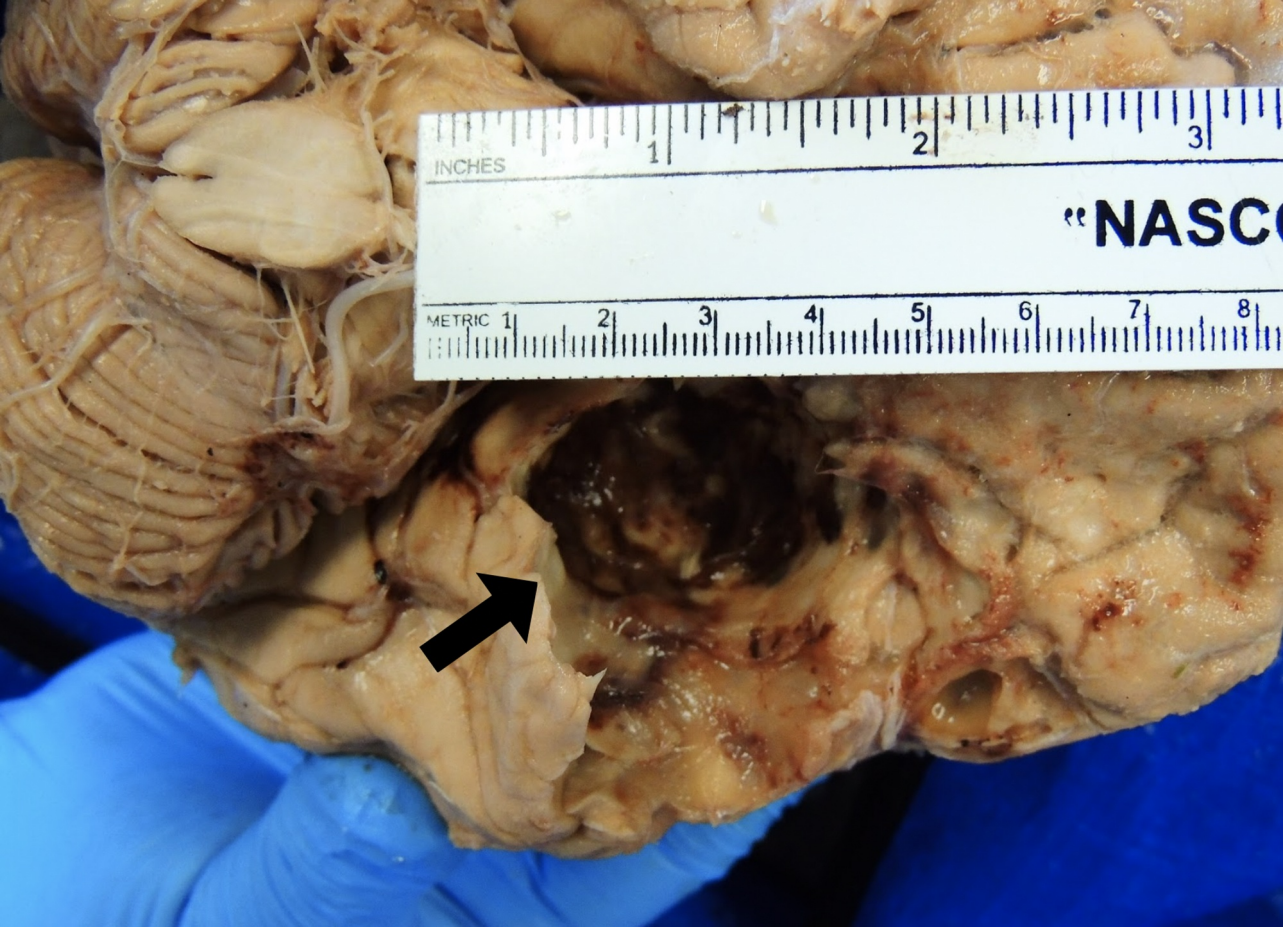
The depression in the left temporal lobe (arrow) caused by the meningioma has an estimated size of 4.5 cm anterior to posterior.

Routine dissection of the cadaver indicated unilateral proptosis of the left eyeball (Figure 1). To investigate the cause of the structural deformity, the scalp was reflected, and evidence of a previous craniotomy was found in the left temporal and parietal bones (Figure 2). Upon removal of the calvaria, the brain parenchyma was carefully examined. Though there was no evidence of tumor invasion into the brain parenchyma, it was clear that the ventral aspect of the left temporal lobe had been severely compressed by the meningioma (Figure 3). The depression formed by the tumor in the temporal lobe had an estimated size of 4.5 cm anterior to posterior (Figure 4). Mild compression of the left frontal lobe was also noted (Figure 3).

**Figure 5:**
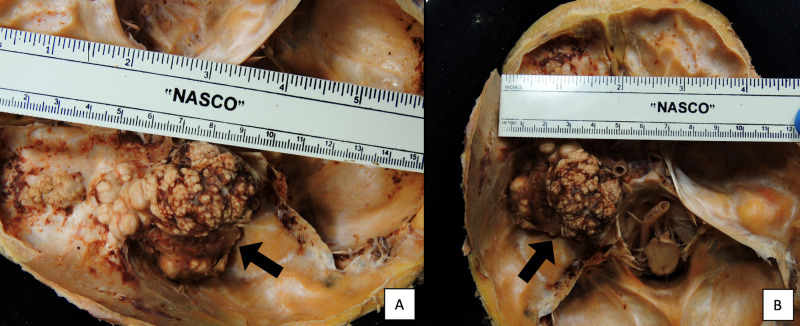
(A) The length of the tumor occupying the middle cranial fossa (arrow) is estimated to be 4.5 cm anterior to posterior. (B) The width of the tumor occupying the middle cranial fossa (arrow) is estimated to be 5 cm.

**Figure 6:**
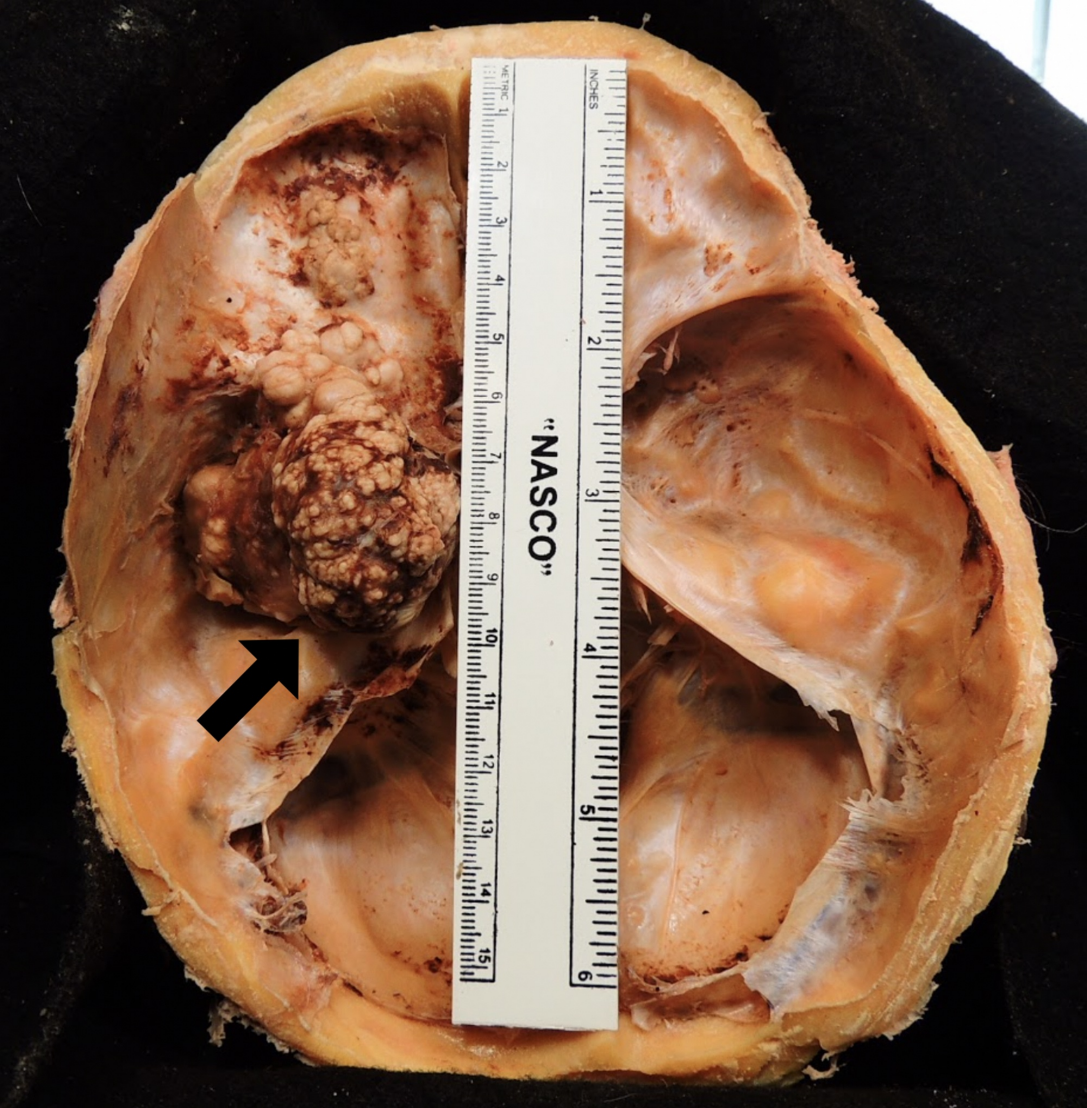
The complete length of the tumor (arrow) occupying the middle and anterior cranial fossae is 9.5 cm.

Examination of the skull base revealed a large cauliflower-like mass extended along the sphenoid wing, nearly filling the left middle cranial fossa. The lobulated tumor appeared brownish-white, had a firm to hard consistency, and had an estimated size of 4.5 cm anterior to posterior and 5 cm in width (Figure 5). The tumor had further extended through the superior orbital fissure and invaded the anterior cranial fossa. The meningioma occupied the left orbit, causing anterior displacement of the left eyeball and subsequent stretching of the left optic nerve. The complete size of the tumor spanning from the middle cranial fossa to the anterior cranial fossa was estimated to be 9.5 cm anterior to posterior (Figure 6).

**Figure 7:**
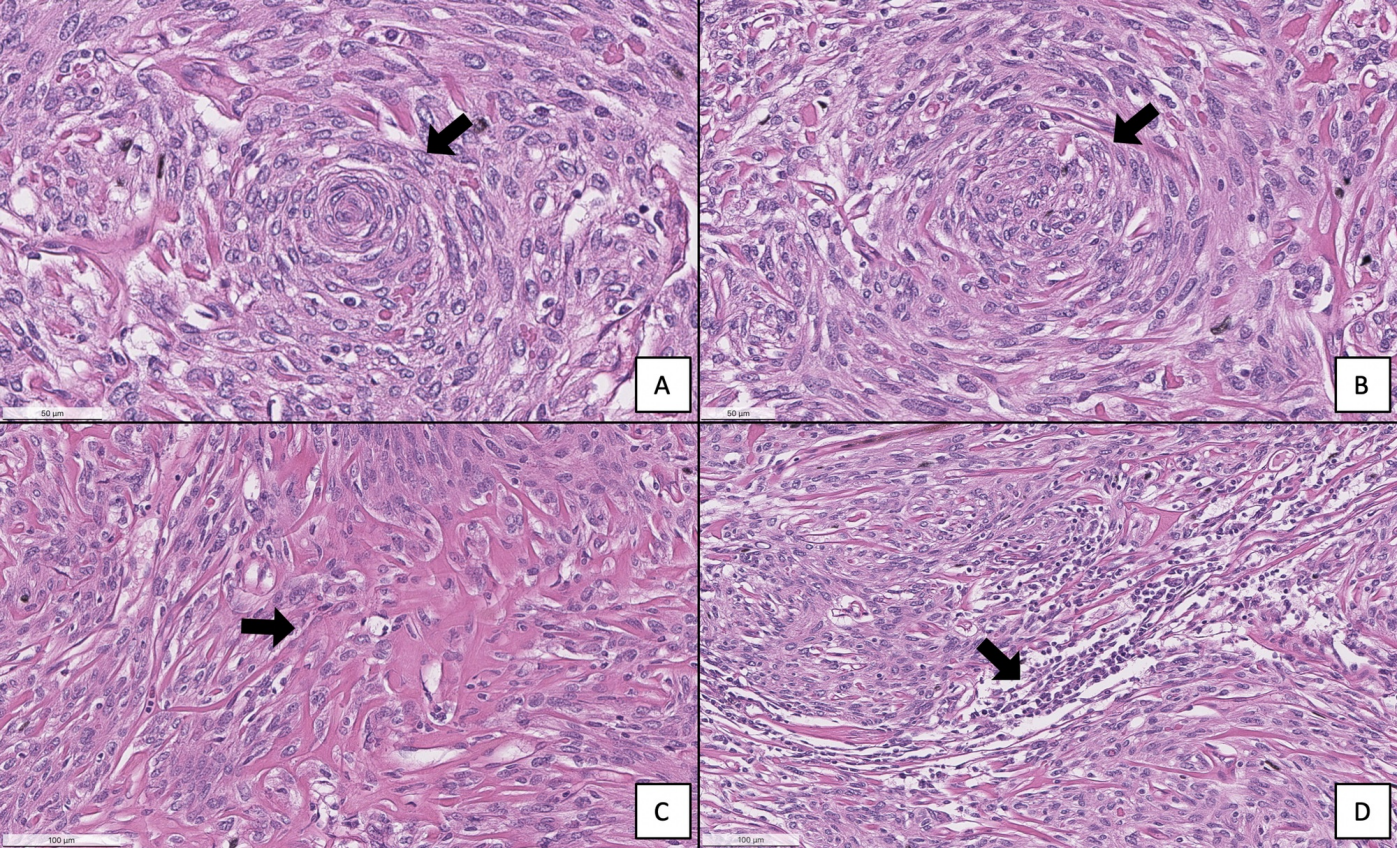
(A, B) A whorled cellular pattern (arrow) is sparsely distributed within the tumor specimen. (C) Collagenous septae (arrow) separate tumor cells. (D) Clusters of dark, dense appearing lymphoplasmocytes (arrow) are distributed throughout the specimen.

**Figure 8:**
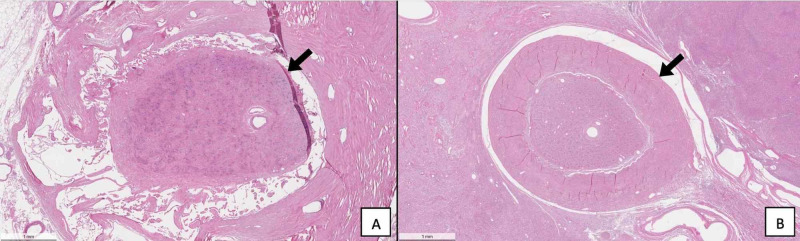
(A) This micrograph displays the unaffected right optic nerve (arrow). (B) The affected left optic nerve displays fewer axons and a thickened optic nerve sheath (arrow).

**Figure 9:**
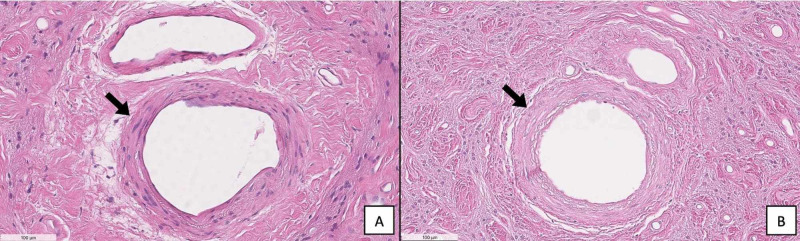
(A) This micrograph displays the unaffected right central retinal artery (arrow). (B) The walls of the left central retinal artery appear acellular (arrow).

Histopathological examination of a section taken from the skull-base tumor revealed a sparse distribution of the whorled cellular pattern that is typical of meningiomas, thin collagenous septae separating tumor cell lobules, and lymphoplasmocytes (Figure 7). No psammoma bodies were found. These features led to the classification of the tumor as a grade I meningothelial meningioma. Histological cross sections of the left and right optic nerves demonstrated that the affected left optic nerve displayed decreased cellularity, loss of axons, and increased connective tissue deposits compared to the normal right optic nerve (Figure 8). The left optic nerve sheath was also thickened as compared to the contralateral nerve. Additionally, the walls of the central retinal artery in the left optic nerve appeared acellular in comparison to those present on the right side (Figure 9).

## Discussion

The meningioma presented in this case study occupied both the left cranium and orbit, compressing the temporal and frontal lobes, thickening the optic nerve sheath, and diminishing axons in the optic nerve (Figures 1, 3, 6, 8). Without a medical history containing CT or MRI images for this cadaver, we are unable to determine if the SOM originated intracranially or intraorbitally. However, within the central nervous system, most meningiomas are reported to originate intracranially, among which common locations include the parasagittal and falx regions (25%), convexities of the brain parenchyma (20%), and the sphenoid ridge (20%) [8]. Intraspinal- and intraorbital-originating meningiomas occur infrequently and have each been reported in less than 2% of all meningioma cases. It is therefore likely that this tumor originated in the left middle cranial fossa and spread anteriorly to invade the orbit.

Although the underlying cause of meningiomas remains unclear, certain genetic factors result in increased susceptibility to develop meningiomas. There was no evidence that suggested that the individual presented in this case suffered from NF2; however, it could be possible that the meningioma resulted from a sporatic mutation in *NF2*. The *NF2* gene encodes for Merlin (moesin-ezrin-radixin-like protein), a cytoskeleton scaffolding protein with tumor suppressor properties [9]. Additionally, Merlin protein expression was not analyzed in this study; however, it is plausible that the meningioma cells exhibited somatic mutations in *NF2*, and therefore had non-functional Merlin. The loss of Merlin results in increased tumor cell proliferation due to loss of contact inhibition mechanisms [9-11]. Although *NF2 *mutations are the most common, somatic mutations on other genes such as *BAM22* and *BCR* on chromosome 22 have also been shown to play a role in meningioma tumorigenesis [12]. Similarly, alterations at loci in other chromosomes have been identified as contributors in the development and progression of meningiomas.

Histological examination and the dural confinement of the neoplasm presented in this case report support its classification as a meningioma. Macroscopically, most meningiomas are well-demarcated, rounded masses that have a characteristic broad dural attachment. In 25%-50% of cases, these tumors will invade through the dura to involve the skull and can cause overproduction, erosion, or infiltration of the bone [13]. However, it is difficult to determine if bone infiltration occurred in this cadaver without radiologic images.

Microscopic examination of the tumor cells confirms it as a grade I meningioma, specifically, the common variant, meningothelial meningioma. Cells forming lobules that are demarcated by thin collagenous septae and oval nuclei with delicate chromatin are characteristics of this subtype (Figure 7C) [2]. While the whorls are not common in this subtype, they can be present and are often less defined compared to other subtypes (Figure 7A). Lastly, the presence of lymphoplasmacytes indicates inflammatory processes that may have occurred within the tumor specimen (Figure 7D).

Grade I meningiomas are typically slow growing and initially asymptomatic. As they grow, they produce varying symptoms depending on compression of adjacent structures through mass effect. Therefore, specific symptoms are subject to the location and size of the tumor. Moreover, headache and seizures are extremely common symptoms of meningiomas in all locations, with epileptic seizures being reported as the first symptom in 20%-50% of meningioma patients [2,4]. Patients with intracranial meningiomas often suffer from disfiguring proptosis, reduced visual acuity, orbital pain, tearing, diplopia, and eyelid swelling [7]. Specifically, those suffering from a meningioma of the lateral sphenoid wing have reported painless unilateral exophthalmos, unilateral loss of vision, and hearing loss [4]. It is clear that the cadaver presented in this case report suffered from proptosis (Figure 1) and likely experienced visual deficits in the left eye due to stretching and compression of the optic nerve as evident from the loss of axons (Figure 8B).

This particular tumor is of interest because of its location and remarkable size. The estimated size of this SOM is 9.5 cm anterior to posterior and 5 cm in width (Figure 6). Magill et al. studied 1,113 patients with meningiomas to examine relationships between location, size, and WHO grade of these neoplasms. The majority (81%) of these patients had WHO grade I tumors. Of the 1,113 meningiomas, the mean tumor size was 3.8 cm and the median size was 3.6 cm. They determined that tumor size was significantly associated with a meningioma being WHO grade II, indicating that the meningioma presented in this case study is uncommonly large for being WHO grade I [14]. Another study conducted by Oya et al. investigated the relationship between diameter measurements and management of meningiomas in 161 patients. They reported that the mean starting meningioma diameter was 20.4 mm (range 3-65 mm) [15]. It is clear after reviewing the literature that the meningioma discovered in this cadaver is one of the largest WHO grade I neoplasms reported.

Based on the remaining evidence of a craniotomy, it is evident that an attempt to debulk the tumor had been made (Figure 2). However, with the lack of a medical history, it cannot be determined whether any of the tumor was indeed resected. Surgery is the standard therapeutic treatment of meningiomas, and excision alone will cure the majority of patients [4]. Typically, excision includes removal of the tumor, dural attachment, and any infiltrated bone, and it allows for histological diagnosis that guides future treatment. Additional treatment options include radiotherapy and hormone therapy, but are typically reserved for atypical, malignant, or reccurent meningiomas [4]. 

Resection of orbit-invading meningiomas is not as straightforward as intracranial meningiomas and depends largely on the patient’s symptoms, degree of cranial nerve involvement, and original diagnosis. One study investigated a group of 47 patients with intracranial meningiomas that spread to the orbit and found that only 50% of the patients underwent macroscopic radical resection. Over 90% of those patients had improved symptoms such as stabilized or improved vision, reduced proptosis, or decreased eye pain [7]. However, the majority of patients with optic nerve sheath meningioma who underwent resection experienced decreased visual acuity and/or damage to additional cranial nerves.

Cannon et al. examined 12 patients who underwent multidisciplinary surgical resection of SOMs. They determined that the indicating factor for surgery was deterioration in visual function. After surgery, 75% of the patients either had a reduction or stabilization of their proptosis, but visual acuity results were mixed. Overall, they expressed that individuals with SOMs may not benefit from surgery, and these patients should be carefully counseled [5].

Additionally, Leroy et al. studied 70 patients with SOMs. The complete resection rate was only 31%, owing to the difficulty of resecting orbit-invading tumors. Preoperatively, 56 patients had proptosis and 86% exhibited significant improvement after surgery. Of the 52 patients who presented with decreased visual acuity, 74% exhibited stable visual acuity, 14% improved visual acuity, and 11% presented with visual acuity deterioration. Finally, 29% of the patients with SOMs experienced tumor recurrence with a median delay of 22 months. Histological sampling was done in 14 of these patients and 10 of the tumors were classified as grade I meningiomas. Additional radiotherapy was beneficial, especially to the patients with grade I tumors [16]. It is evident that meningiomas that invade the orbit pose additional risks to surgical intervention, and each case should be carefully examined.

## Conclusions

This case report describes a 93-year-old female cadaver presenting with a SOM occupying the middle cranial fossa, extending anteriorly through the superior orbital fissure, and invading the orbital cavity. Such tumors are accompanied by symptoms such as proptosis, decreased visual acuity, diplopia, and orbital pain. Histological analysis revealed that this tumor is a WHO grade I meningothelial meningioma. It grew to a large size of 9.5 cm anterior to posterior and 5 cm wide, which is rare for grade I meningiomas. Surgical excision is the mainstay of treatment for meningiomas and will often result in complete resection and resolution of symptoms. However, orbit-invading meningiomas that involve the optic nerve are difficult to completely resect and can lead to worsened visual acuity and damage to additional cranial nerves. Therefore, it is imperative to assess each case carefully and counsel patients accordingly. The tumor discussed in this case is of interest due to the rarity of such a massive SOM presenting in the ninth decade of life. It is necessary to report these new findings to add to the current knowledge base and to better understand SOM behavior.
